# Budd-Chiari Syndrome: An Unusual Complication of AL Amyloidosis

**DOI:** 10.4274/tjh.galenos.2020.2019.0186

**Published:** 2020-05-06

**Authors:** Tarık Onur Tiryaki, İpek Yönal Hindilerden, Gülçin Yegen, Meliha Nalçacı

**Affiliations:** 1 İstanbul University İstanbul Medical Faculty, Department of Internal Medicine, Division of Hematology, İstanbul, Turkey; 2 İstanbul University İstanbul Medical Faculty, Department of Pathology, İstanbul, Turkey

**Keywords:** Primary amyloidosis, Budd-Chiari syndrome, Plasma cell diseases

## To the Editor,

Budd-Chiari syndrome (BCS) is an uncommon congestive hepatopathy caused by blockage of hepatic veins in the absence of cardiac/pericardial disorders as well as hepatic veno-occlusive disease [1]. In 75% of patients with BCS there is an underlying condition that predisposes to blood clotting [2]. More than one etiological factor may play a role in 25% of cases [2]. Coagulation problems mainly involving bleeding abnormalities are well recognized in AL amyloidosis while thrombosis is a less common feature [3]. Here, we report a rare case of AL amyloidosis complicated by BCS. 

**Figure 1:**
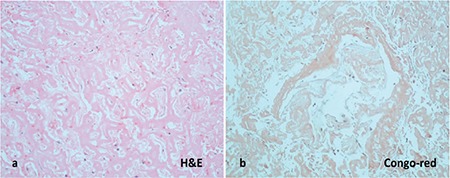
Diffuse infiltration of eosinophilic amorphous material in the liver parenchyma (a, H&E, 400^x^), and deposition positive for Congo red staining (b, 400^x^).

**Figure 2:**
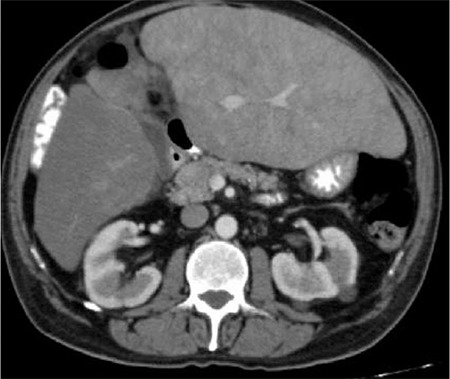
Abdominal CT angiography demonstrated occlusion of the left hepatic vein and enlargement in the left lobe of the liver.

A 66-year-old man presented with right upper abdominal pain. On physical examination, there was hepatomegaly measuring 6 cm below the costal margin. His complete blood count (CBC) was as follows: hemoglobin (Hb), 11 g/dL; white blood cell count, 15,100/mm^3^; neutrophils, 8400/mm^3^; lymphocytes, 4700/mm^3^; platelets, 677,000/mm^3^. The following biochemical tests were abnormal: corrected calcium, 10.74 mg/dL (normal range: 8.5-10.5); albumin, 3.12 g/dL (normal=3.2-5.5); alkaline phosphatase, 266 IU/L (normal=35-105); gamma-glutamyl transferase, 388 IU/L (normal=5-85); C-reactive protein, 26 (normal=0-5); erythrocyte sedimentation rate, 85 mm/h (normal=0-20). JAK2V617F mutation was not detected and bcr-abl was negative. Upon serum protein electrophoresis, a monoclonal protein of 0.01 g/dL was present and serum and urine immunofixation electrophoresis showed monoclonal λ light chain. Serological tests for hepatitis B, hepatitis C, HIV, and autoimmune liver disorders were negative. The result of the 24-h urine protein was 150 mg. Abdominal ultrasonography showed hepatomegaly measuring 189 mm on the longitudinal axis. Liver biopsy showed diffuse amyloid deposits in the parenchyma stained by Congo red (Figure 1). Bone marrow biopsy demonstrated increased plasma cells constituting 20% of the marrow cellularity and eosinophilic, homogeneous deposits of amyloid confirmed by Congo red staining. Echocardiography showed thickened interventricular septum measuring 15 mm. Histological examination of the duodenum revealed amorphous pink deposits in the lamina propria staining positive for Congo red. The patient did not meet the diagnostic criteria for myeloma and was diagnosed with AL amyloidosis with kidney, heart, liver, and gastrointestinal tract involvement. CyBorD was initiated as induction treatment. After 1 course of CyBorD, his CBC results were completely normal. After the 4^th^ course, the patient presented with severe acute right upper quadrant abdominal pain and severe orthostatic hypotension. Abdominal CT angiography showed thrombosis of the left and middle hepatic veins. Intrahepatic venous collaterals and a relative increase in the caudate and left lobes of the liver were noted (Figure 2). These findings were compatible with BCS. Screening for hereditary and/or acquired thrombophilic conditions were negative. Anticoagulation with low-molecular-weight heparin was initiated.

To our knowledge, this is the first reported case of AL amyloidosis complicated by BCS in the absence of nephrotic syndrome. The underlying causes of bleeding in AL amyloidosis are well established, including acquired factor X deficiency, increased intravascular coagulation and fibrinolysis, and capillary infiltration by amyloid and liver involvement, which results in the reduced synthesis of procoagulant proteins [4,5]. Thrombosis is a less-recognized association of AL amyloidosis. It was demonstrated that impairment of the thrombin-antithrombin pathway, in association with low antithrombin biological activity, contributed to  hypercoagulability in amyloidosis [5]. Cançado et al. [6] described a BCS patient diagnosed with AL amyloidosis in the concomitant presence of nephrotic-range proteinuria. The loss of hemostatic proteins due to nephrotic syndrome certainly contributed to the imbalance between clotting factors and inhibitors [6]. Although arterial thrombosis after bortezomib treatment has been reported rarely, a review of data from phase 3 trials demonstrated lower venous thromboembolism risk with bortezomib [7,8]. Therefore, we believe that there is no association between BCS and bortezomib. Our case shows that AL amyloidosis patients can develop BCS even in the absence of nephrotic syndrome.
